# Seven Pervasive Statistical Flaws in Cognitive Training Interventions

**DOI:** 10.3389/fnhum.2016.00153

**Published:** 2016-04-14

**Authors:** David Moreau, Ian J. Kirk, Karen E. Waldie

**Affiliations:** Centre for Brain Research and School of Psychology, University of AucklandAuckland, New Zealand

**Keywords:** brain enhancement, evidence-based interventions, working memory training, intelligence, methods, data analysis, statistics, experimental design

## Abstract

The prospect of enhancing cognition is undoubtedly among the most exciting research questions currently bridging psychology, neuroscience, and evidence-based medicine. Yet, convincing claims in this line of work stem from designs that are prone to several shortcomings, thus threatening the credibility of training-induced cognitive enhancement. Here, we present seven pervasive statistical flaws in intervention designs: (i) lack of power; (ii) sampling error; (iii) continuous variable splits; (iv) erroneous interpretations of correlated gain scores; (v) single transfer assessments; (vi) multiple comparisons; and (vii) publication bias. Each flaw is illustrated with a Monte Carlo simulation to present its underlying mechanisms, gauge its magnitude, and discuss potential remedies. Although not restricted to training studies, these flaws are typically exacerbated in such designs, due to ubiquitous practices in data collection or data analysis. The article reviews these practices, so as to avoid common pitfalls when designing or analyzing an intervention. More generally, it is also intended as a reference for anyone interested in evaluating claims of cognitive enhancement.

## Introduction

Can cognition be enhanced via training? Designing effective interventions to enhance cognition has proven one of the most promising and difficult challenges of modern cognitive science. Promising, because the potential is enormous, with applications ranging from developmental disorders to cognitive aging, dementia, and traumatic brain injury rehabilitation. Yet difficult, because establishing sound evidence for an intervention is particularly challenging in psychology: the gold standard of double-blind randomized controlled experiments is not always feasible, due to logistic shortcomings or to common difficulties in disguising the underlying hypothesis of an experiment. These limitations have important consequences for the strength of evidence in favor of an intervention. Several of them have been extensively discussed in recent years, resulting in stronger, more valid, designs.

For example, the importance of using active control groups has been underlined in many instances (e.g., Boot et al., 2013), helping conscientious researchers move away from the use of no-contact controls, standard in a not-so-distant past. Equally important is the emphasis on objective measures of cognitive abilities rather than self-report assessments, or on the necessity to use multiple measurements of single abilities to provide better estimates of cognitive constructs and minimize measurement error (Shipstead et al., 2012). Other limitations pertinent to training designs have been illustrated elsewhere with simulations (e.g., fallacious assumptions, Moreau and Conway, 2014; biased samples, Moreau, 2014b), in an attempt to illustrate visually some of the discrepancies observed in the literature. Indeed, much knowledge can be gained by incorporating simulated data to complex research problems (Rubinstein and Kroese, 2011), either because they are difficult to visualize or because the representation of their outcomes is ambiguous. Intervention studies are no exception—they often include multiple extraneous variables, and thus benefit greatly from informed estimates about the respective influence of each predictor variable in a given model. As it stands, the approach typically favored is that good experimental practices (e.g., random assignment, representative samples) control for such problems. In practice, however, numerous designs and subsequent analyses do not adequately allow such inferences, due to single or multiple flaws. We explore here some of the most prevalent of these flaws.

Our objective is three-fold. First, we aim to bring attention to core methodological and statistical issues when designing or analyzing training experiments. Using clear illustrations of how pervasive these problems are, we hope to help design better, more potent interventions. Second, we stress the importance of simulations to improve the understanding of research designs and data analysis methods, and the influence they have on results at all stages of a multifactorial project. Finally, we also intend to stimulate broader discussions by reaching wider audiences, and help individuals or organizations assess the effectiveness of an intervention to make informed decisions in light of all the evidence available, not just the most popular or the most publicized information. We strive, throughout the article, to make every idea as accessible as possible and to favor clear visualizations over mathematical jargon.

A note on the structure of the article. For each flaw we discuss, we include three steps: (1) a brief introduction to the problem and a description of its relation to intervention designs; (2) a Monte Carlo simulation and its visual illustration^1^; and (3) advice on how to circumvent the problem or minimize its impact. Importantly, the article is not intended to be an in-depth analysis of each flaw discussed; rather, our aim is to help visual representations of each problem and provide the tools necessary to assess the consequences of common statistical procedures. However, because the problems we discuss hereafter are complex and deserve further attention, we have referred the interested reader to additional literature throughout the article.

A slightly more technical question pertains to the use of Monte Carlo simulations. Broadly speaking, Monte Carlo methods refer to the use of computational algorithms to simulate repeated random sampling, in order to obtain numerical estimates of a process. The idea that we can refine knowledge by simulating stochastic processes repeatedly rather than via more traditional procedures (e.g., direct integration) might be counterintuitive, yet this method is well suited to the specific examples we are presenting here for a few reasons. Repeated stochastic simulations allow creating mathematical models of ecological processes: the repetition represents research groups, throughout the world, randomly sampling from the population and conducting experiments. Such simulations are also particularly useful in complex problems where a number of variables are unknown or difficult to assess, as they can provide an account of the values a statistic can take when constrained by initial parameters, or a range of parameters. Finally, Monte Carlo simulations can be clearly represented visually. This facilitates the graphical translation of a mathematical simulation, thus allowing a discussion of each flaw with little statistical or mathematical background.

## Lack of Power

We begin our exploration with a pervasive problem in almost all experimental designs, particularly in training interventions: low statistical power. In a frequentist framework, two types of errors can arise at the decision stage in a statistical analysis: Type I (false positive, probability α) and Type II (false negative, probability β). The former occurs when the null hypothesis (H_0_) is true but rejected, whereas the latter occurs when the alternative hypothesis (H_A_) is true but the H_0_ is retained. That is, in the context of an intervention, the experimental treatment was effective but statistical inference led to the erroneous conclusion that it was not. Accordingly, the power of a statistical test is the probability of rejecting H_0_ given that it is false. The more power, the lower the probability of Type II errors, such that power is (1−β). Importantly, higher statistical power translates to a better chance of detecting an effect if it is exists, but also a better chance that an effect is genuine if it is significant (Button et al., 2013). Obviously, it is preferable to minimize β, which is akin to maximizing power.

Because α is set arbitrarily by the experimenter, power could be increased by directly increasing α. This simple solution, however, has an important pitfall: since α represents the probability of Type I errors, any increase will produce more false positives (rejections of H_0_ when it should be retained) in the long run. Therefore, in practice experimenters need to take into account the tradeoff between Type I and Type II errors when setting α. Typically, α < β, because missing an existing effect (β) is thought to be less prejudicial than falsely rejecting H_0_ (α); however, specific circumstances where the emphasis is on discovering new effects (e.g., exploratory approaches) sometimes justify α increases (for example, see Schubert and Strobach, 2012).

**Figure 1:**
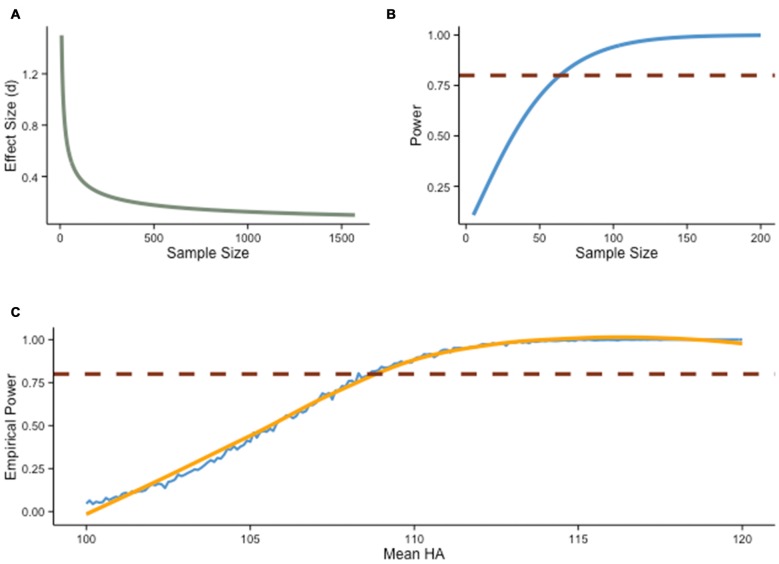
**Power analysis. (A)** Relationship between effect size (Cohen’s *d*) and sample size with power held constant at .80 (two-sample *t*-test, two-sided hypothesis, α = .05). **(B)** Relationship between power and sample size with effect size held constant *d* = 0.5 (two-sample *t*-test, two-sided hypothesis, α = .05). **(C)** Empirical power as a function of an alternative mean score (H_A_), estimated from a Monte Carlo simulation (*N* = 1000) of two-sample *t*-tests with 20 subjects per group. The distribution of scores under null hypothesis (H_0_) is consistent with IQ test score standards (*M* = 100, SD = 15), and H_A_ varies from 100 to 120 by incremental steps of 0.1. The blue line shows the result of the simulation, with estimated smoothed curve (orange line). The red line denotes power = .80.

Discussions regarding experimental power are not new. Issues related to power have long been discussed in the behavioral sciences, yet they have drawn heightened attention recently (e.g., Button et al., 2013; Wagenmakers et al., 2015), for good reasons: when power is low, relevant effects might go undetected, and significant results often turn out to be false positives^2^. Besides α and β levels, power is also influenced by sample size and effect size (Figure 1A). The latter depends on the question of interest and the design, with various strategies intended to maximize the effect one wishes to observe (e.g., well-controlled conditions). Noise is often exacerbated in training interventions, because such designs potentially increase sources of non-sampling errors, for example via poor retention rates, failure to randomly assigned participants, use of non-standardized tasks, use of single measures of abilities, or failure to blind participants and experimenters. Furthermore, the influence of multiple variables is typically difficult to estimate (e.g., extraneous factors), and although random assignment is usually thought to control for this limitation, it has been demonstrated repeatedly that such assumption is highly dependent on sample size, with typical designs being rarely satisfactory in this regard (Cohen, 1992b). As a result, the preferred solution to increase power is typically to adjust sample sizes (Figure 1B).

Power analyses are especially relevant in the context of interventions because sample size is usually limited by the design and its inherent costs—training protocols require participants to come back to the laboratory multiple times for testing and in some cases for the training regimen itself. Yet despite the importance of precisely determining power before an experiment, power analyses include several degrees of freedom that can radically change outcomes and thus recommended sample sizes (Cohen, 1992b). As informative as it may be, gauging the influence of each factor is difficult using power analyses in the traditional sense, that is, varying factors one at a time. This problem can be circumvented by Monte Carlo methods, where one can visualize the influence of each factor in isolation and in conjunction with one another.

Suppose, for example, that we wish to evaluate the effectiveness of an intervention by comparing gain scores in experimental and control groups. Using a two-sample *t*-test with two groups of 20 subjects, and assuming α = .05 and 1−β = .80, an effect size needs to be of about *d* = 0.5 or greater to be detected, on average (Figure 1C). Any weaker effect would typically go undetected. This concern is particularly important when considering how conservative our example is: a power of .80 is fairly rare in typical training experiments, and an effect size of *d* = 0.5 is quite substantial—although typically defined as “medium” in the behavioral sciences (Cohen, 1988), an increase of half a standard deviation is particularly consequential in training interventions, given potential applications and the inherent noise of such studies.

We should emphasize that we are not implying that every significant finding with low power should be discarded; however, caution is warranted when underpowered studies coincide with unlikely hypotheses, as this combination can lead to high rates of Type I errors (Krzywinski and Altman, 2013; Nuzzo, 2014). Given the typical lack of power in the behavioral sciences (Cohen, 1992a; Button et al., 2013), the current emphasis on replication (Pashler and Wagenmakers, 2012; Baker, 2015; Open Science Collaboration, 2015) is an encouraging step, as it should allow extracting more signal from noisy, underpowered experiments in the long run. Statistical power directly informs the reader about two elements: if an effect is there, what is the probability to detect it, and if an effect was detected, what is the probability that it was genuine? These are critical questions in the evaluation of scientific evidence, and especially in the field of cognitive training, setting the stage for the central role of power in all the problems discussed henceforth.

## Sampling Error

**Figure 2:**
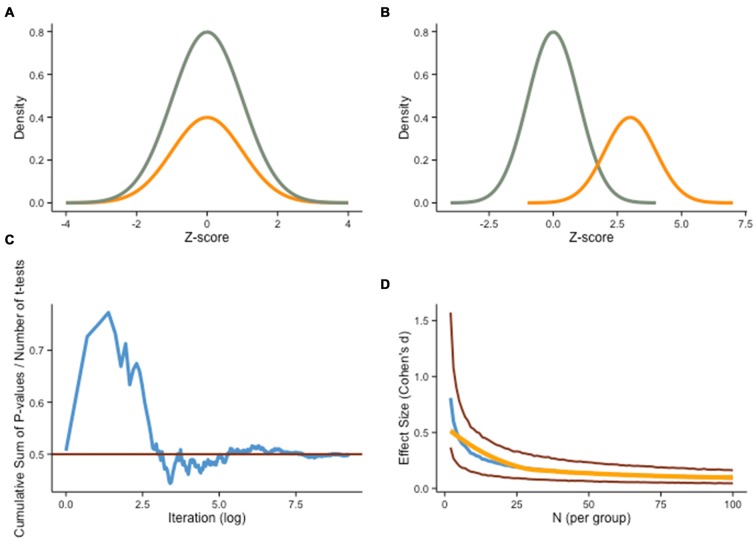
**Sampling error. (A)** Cognitive training experiments often assume a theoretical ideal where the sample (orange curve) is perfectly representative of the true underlying distribution (green curve). **(B)** However, another possibility is that the sample is not representative of the population of interest due to sampling error, a situation that can lead to dubious claims regarding the effectiveness of a treatment. **(C)** Assuming α = .05 in a frequentist framework, the difference between two groups drawn from the same underlying distribution will be unequal 5% of the time, but a more subtle departure from the population is also likely to influence training outcomes meaningfully. The graph shows the cumulative sum of two-sample *t*-test *p*-values divided by the number of tests performed, based on a Monte Carlo simulation (*N* = 10,000) of 40 individual IQ scores (normally distributed, *M* = 100, SD = 1) randomly divided in two groups (experimental, control). The red line shows *P* = .5. **(D)** Even when both groups are drawn from a single normal distribution (H_0_ is true), small sample sizes will spuriously produce substantial differences (absolute median effect size, blue line), as illustrated here with another Monte Carlo simulation (*N* = 10,000). As group samples get larger, effect size estimates get closer to 0. Red lines represent 25th and 75th quartiles, and the orange line is loess smooth on the median.

A pernicious consequence of low statistical power is sampling error. Because a sample is an approximation of the population, a point estimate or statistic calculated for a specific sample may differ from the underlying parameter in the population (Figures 2A,B). For this reason, most statistical procedures take into account sampling error, and experimenters try to minimize its impact, for example by controlling confounding factors, using valid and reliable measures, and testing powerful manipulations. Despite these precautions, sampling error can obscure experimental findings in an appreciable number of occurrences (Schmidt, 1992). We provide below an example of its detrimental effect.

Let us consider a typical scenario in intervention designs. Assume we randomly select a sample of 40 individuals from an underlying population and assign each participant either to the experimental or the control group. We now have 20 participants in each group, which we assume are representative of the whole population. This assumption, however, is rarely met in typical designs (e.g., Campbell and Stanley, 1966). In small samples, sampling error can have important consequences, especially when individual characteristics are not homogeneously represented in the population. Differences can be based upon neural plasticity, learning potential, motivational traits, or any other individual characteristic. When sampling from an heterogeneous population, groups might not be matched despite random assignment (e.g., Moreau, 2014b).

In addition, failure to take into account extraneous variables is not the only problem with sampling. Another common weakness relates to differences in pretest scores. As set by α, random sampling will generate significantly different baseline scores on a given task 5% of the time in the long run, despite drawing from the same underlying population (see Figure 2C). This is not trivial, especially considering that less sizeable discrepancies can significantly influence the outcome of an intervention, as training or testing effects might exacerbate a difference undetected initially.

There are different ways to circumvent this problem, and one in particular that has been the focus of attention recently in training interventions is to increase power. As we have mentioned in the previous section, this can be accomplished either by using larger samples, or by studying larger effects, or both (Figure 2D). But these adjustments are not always feasible. To restrict the influence of sampling error, another potential remedy is to factor pretest performance on the dependent variable into group allocation, via restricted randomization. The idea is to ensure that random assignment has been effective at shuffling predefined characteristics (e.g., scores, demographics, physiological correlates) evenly to the different experimental conditions. If groups are imbalanced, a simple remedy is to perform new iterations of the random assignment procedure until conditions are satisfied. This is sometimes unpractical, however, especially with multiple variables to shuffle. Alternatively, one can then constrain random assignment *a priori* based on pretest scores, via stratified sampling (e.g., Aoyama, 1962). Non-random methods of group assignment are sometimes used in training studies (Spence et al., 2009; Loosli et al., 2012; Redick et al., 2013). An example of such methods, blocking, consists of dividing participants based on pretest scores on a given variable, to create homogenous groups (Addelman, 1969). In second step, random assignment is performed with equal draws from each of the groups, so as to preserve the initial heterogeneity in each experimental group. Other, more advanced approaches can be used (for a review, see Green et al., 2014), yet the rationale remains the same, that is, to reduce the influence of initial discrepancies on the outcome of an intervention. We should point out that these procedures bring problems of their own (Ericson, 2012)—with small samples, no method of assignment is perfect, and one needs to decide on the most suitable approach based on the specific design and hypotheses. In an effort to be transparent, it is therefore important to report how group assignment was performed, particularly in instances where it departed from typical (i.e., simple) randomization.

## Continuous Variable Splits

Lack of power and its related issue sampling error are two limitations of experimental designs that often need substantial investment to be remediated. Conversely, splitting a continuous variable is a deliberate decision at the analysis stage. Although popular in intervention studies, it is rarely—if ever—justified.

Typically, a continuous variable reflecting performance change throughout training is split into a categorical variable, often dichotomous. Because the idea is to identify individuals who do respond to the training regimen, and those who do not benefit as much, this approach is often called “responder analysis”. Most commonly, the dichotomization is achieved via a median split, which refers to the procedure of finding the median score on a continuous variable (e.g., training performance) and split subjects who are below and above this particular score (e.g., low responders vs. high responders).

Median splits are almost always prejudicial (Cohen, 1983), and their use often reflects a lack of understanding of the consequences involved (MacCallum et al., 2002). A full account of the problems associated with this practice is beyond the scope of this article, but the main harms are loss of power and of information, reduction of effect sizes, and inconsistencies in the comparison of results across studies (Allison et al., 1993). Turning a continuous variable into a dichotomy also implies that the original continuum was irrelevant, and that the true nature of the variable is dichotomous. This is seldom the case.

In intervention designs, a detrimental consequence of turning continuous variables into categorical ones and separating low and high performers *post hoc* is the risk of regression toward the mean (Galton, 1886). Regression toward the mean is one of the most well known byproducts of multiple measurements, yet it is possibly one of the least understood (Nesselroade et al., 1980). As for all the notions discussed in this article, regression toward the mean is not exclusive to training experiments; however, estimating its magnitude is made more difficult by potential confounds with testing effects in these types of design.

In short, regression toward the mean is the tendency for a given observation that is extreme, or far from the mean, to be closer to the mean on a second measurement. When a population is normally distributed, extreme scores are not as likely as average scores, therefore making the probability to observe two extreme scores in a row unlikely. Regression toward the mean is the consequence of imperfect correlations between scores from one session to the next—singling out an extreme score on a specific measure therefore increases the likelihood that it will regress to the mean on another measurement.

**Figure 3:**
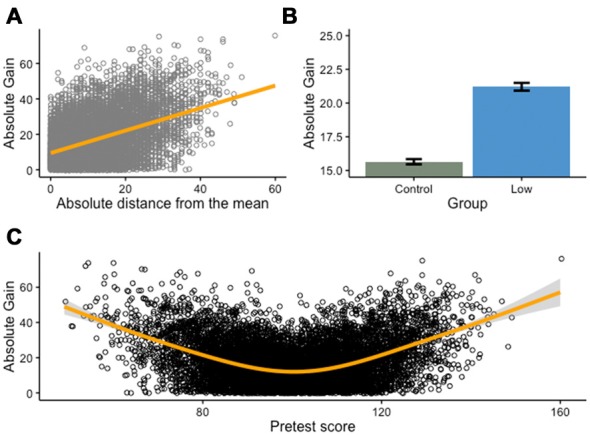
**Continuous variable splits and regression toward the mean.** Many interventions isolate a low-performing group at pretest and compare the effect of training on this group with a group that includes the remaining participants. This approach is fundamentally flawed, as it capitalizes on regression toward the mean rather than on true training effects. Here, we present a Monte Carlo simulation of 10,000 individual scores drawn from a normally-distributed population (*M* = 100, SD = 15), before and after a cognitive training intervention (for simplicity purposes, we assume no test-retest effect). **(A)** As can be expected in such a situation, there is a positive relationship between the absolute distance of pretest scores from the mean and the absolute gains from pretest to posttest: the farther a score deviates from the mean (in either direction), the more likely it is to show important changes between the two measurement points. **(B)** This is particularly problematic when one splits a continuous variable (e.g., pretest score) into a categorical variable. In this case, and without assuming any real effect of the intervention, a low-performing group (all scores inferior or equal to the first quartile) will typically show impressive changes between the two measurement points, compared with a control group (remaining scores). **(C)** This difference is a direct consequence of absolute gains from pretest to posttest being a function of the deviation of pretest scores from the mean, following a curvilinear relationship (U-shaped, orange line).

This phenomenon might be puzzling because it seems to violate the assumption of independent events. Indeed, regression toward the mean can be mistaken as a deterministic linear change from one measurement to the next, whereas it simply reflects the idea that in a bivariate distribution with the correlation between two variables *X* and *Y* less that |1|, the corresponding value *y* in *Y* of a given value *x* of *X* is expected to be closer to the mean of *Y* than *x* is to the mean of *X*, provided both are expressed in standard deviation units (Nesselroade et al., 1980). This is easier to visualize graphically—the more a score deviates from the mean on a measurement (Figure 3A), the more it will regress to the mean on a second measurement, independently from any training effect (i.e., assuming no improvement from pretest to posttest). This effect is exacerbated after splitting a continuous variable (Figure 3B), as absolute gains are influenced by the deviation of pretest scores from the mean, irrespective of genuine improvement (Figure 3C).

This is particularly problematic in training interventions because numerous studies are designed to measure the effectiveness of a treatment after an initial selection based on baseline scores. For example, many studies intend to assess the impact of a cognitive intervention in schools after enrolling the lowest-scoring participants on a pretest measure (e.g., Graham et al., 2007; Helland et al., 2011; Stevens et al., 2013). Median- or mean-split designs should always wary the reader, as it does not adequately control for regression toward the mean and other confounds (e.g., sampling bias) – if the groups to be compared are not equal at baseline, any interpretation of improvement is precarious. In addition, such comparison is often obscured by the sole presentation of gains scores, rather than both pretest and posttest scores. Significant gains in one group vs. the other might be due to a true effect of the intervention, but can also arise from unequal baseline scores. The remedy is simple: unless theoretically motivated *a priori*, splitting a continuous variable should be avoided, and unequal performance at baseline should be reported and taken into account when assessing the evidence for an intervention.

Despite the questionable relevance of this practice, countless studies have used median splits on training performance scores in the cognitive training literature (Jaeggi et al., 2011; Rudebeck et al., 2012; Kundu et al., 2013; Redick et al., 2013; Thompson et al., 2013; Novick et al., 2014), following the rationale that transfer effects are moderated by individual differences in gains on the training task (Tidwell et al., 2014). Accordingly, individual differences in response to training and cognitive malleability leads researchers to expect a correlation between training gains and gains on the transfer tasks, a finding that has been commonly reported in the literature (Chein and Morrison, 2010; Jaeggi et al., 2011; Schweizer et al., 2013; Zinke et al., 2014). We explore this idea further in the next section.

## Interpretation of Correlations in Gains

The goal in most training interventions is to show that training leads to transfer, that is, gains in tasks that were not part of the training. Decades of research have shown that training on a task results in enhanced performance on this particular task, paving the way for entire programs of research focusing on deliberate practice (e.g., Ericsson et al., 1993). In the field of cognitive training, however, the newsworthy research question is whether or not training is followed by enhanced performance on a *different* task (i.e., transfer). Following this rationale, researchers often look for positive correlations between gains in the training task and in the transfer task, and interpret such effects as evidence supporting the effectiveness of an intervention (Jaeggi et al., 2011; Rudebeck et al., 2012; Kundu et al., 2013; Redick et al., 2013; Thompson et al., 2013; Novick et al., 2014; Zinke et al., 2014). Although apparently sound, this line of reasoning is flawed. Correlated gain scores are neither an indication nor a necessity for transfer—transfer can be obtained without any correlation in gain scores, and correlated gain scores do not guarantee transfer (Zelinski et al., 2014).

**Figure 4:**
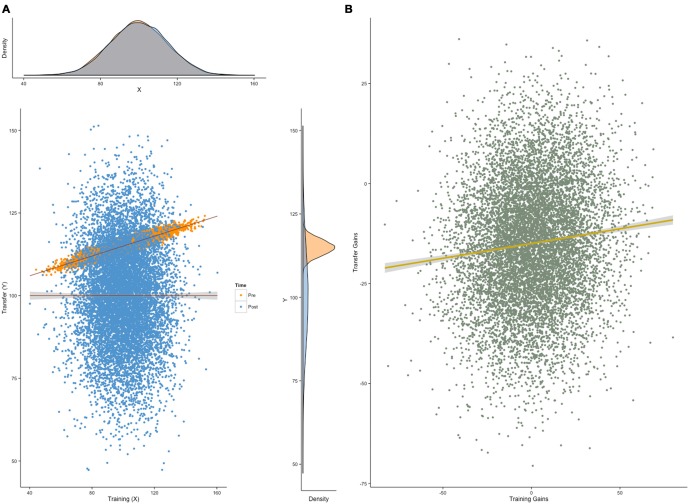
**Correlated gain scores.** Correlated gain scores between a training variable and a transfer variable can occur regardless of transfer. **(A)** Here, a Monte Carlo simulation (*N* = 1000) shows normally-distributed individual scores (*M* = 100, SD = 15) on a training task and a transfer task at two consecutive testing sessions (pretest in orange, posttest in blue). The only constraint on the model is the correlation between the two tasks at pretest (here, *r* = .92), but not at posttest. **(B)** In this situation, gain scores—defined as the difference between posttest and pretest scores—will be correlated (here, *r* = .10), regardless of transfer. This pattern can arise irrespective of training effectiveness, due to the initial correlation between training and transfer scores.

For the purpose of simplicity, suppose we design a training intervention in which we set out to measure only two dependent variables: the ability directly trained (e.g., working memory capacity, WMC) and the ability we wish to demonstrate transfer to (e.g., intelligence, *g*). If requirements (a, b, c) are met such that: (a) performance on WMC and *g* is correlated at pretest, as is often the case due to the positive manifold (Spearman, 1904), (b) this correlation is no longer significant at posttest, and (c) scores at pretest do not correlate well with scores at posttest, both plausible given that one ability is being artificially inflated through training (Moreau and Conway, 2014); then gains in the trained ability and in the transfer ability will be correlated. This correlation will be a consequence of pretest correlations, and cannot be regarded as reflecting evidence for transfer. More strikingly perhaps, performance gains in initially correlated tasks are expected to be correlated even *without* transfer (Figures 4A,B). Correlations are unaffected by a linear transformation of the variables they relate to—they are therefore not influenced by variable means. As a result, correlated gain scores is a phenomenon completely independent from transfer. A positive correlation is the consequence of a greater covariance of gain scores within-session than between sessions, but it provides no insight into the behavior of the means we wish to measure—scores could increase, decrease, or remain unchanged, and this information would not be reflected in the correlation of gain scores (Tidwell et al., 2014). Conversely, transfer can happen without correlated gains, although this situation is perhaps less common in training studies, as it often implies that the training task and the transfer task were not initially correlated.

To make things worse, analyses of correlation in gains are often combined with median splits to look for different patterns in a group of responders (i.e., individuals who improved on the training task) and in a group of non-responders (i.e., individuals who did not improve on the training task). The underlying rationale is that if training is effective, only those who improved in the training task should show transfer. This approach, however, combines the flaw we presented herein with the ones discussed in the previous section, therefore increasing the chances to reach erroneous conclusions. Limitations of this approach have been examined before and illustrated via simulations (Tidwell et al., 2014) and structural equation modeling (SEM; Zelinski et al., 2014). To summarize, these articles point out that correlated gain scores do not answer the question they are typically purported to answer, that is, whether improvement was moderated by training conditions.

The remedy to this intuitive but erroneous interpretation of correlated gains lies in alternative statistical techniques. Transfer can be established when the experimental group shows larger gains than controls, demonstrated by a significant interaction on a repeated measures ANOVA (with treatment group as the between-subject factor and session as the within-group factor) or its Bayesian analog. Because this analysis does not correct for group differences at pretest, one should always report *post hoc* comparisons to follow up on significant interactions and provide summary statistics including pretest and posttest scores, not just of gain scores, as is often the case. Due to this limitation, another common approach is to use an ANCOVA, with posttest scores as a dependent variable and pretest scores as a covariate. Although often used interchangeably, the two types of analysis actually answer slightly different research questions. When one wishes to assess the difference in gains between treatment groups, the former approach is most appropriate^3^. Unlike correlated gain scores, this method allows answering the question at hand—does the experimental treatment produce larger cognitive gains than the control?

A different, perhaps more general problem concerns the validity of improvements typically observed in training studies. How should we interpret gains on a specific task or on a cognitive construct? Most experimental tasks used by psychologists to assess cognitive abilities were designed and intended for comparison between individuals or groups, rather than as a means to quantify individual or group improvements. This point may seem trivial, but it hardly is—the underlying mechanisms tapped by training might be task-specific, rather than domain-general. In other words, one might improve via specific strategies that help perform well on a task or set of tasks, without any guarantee of meaningful transfer. In some cases, even diminishment can be viewed as a form of enhancement (Earp et al., 2014). It can therefore be difficult to interpret improvement following a training intervention, as it may reflect different underlying patterns. Hayes et al. (2015, p. 1) emphasize this point in a discussion of training-induced gains in fluid intelligence: “The interpretation of these results is questionable because score *gains* can be dominated by factors that play marginal roles in the scores themselves, and because intelligence gain is not the only possible explanation for the observed control-adjusted far transfer across tasks”. Indeed, a possibility that often cannot be discarded is that improvement is driven by strategy refinement rather than general gains. Moreover, it has also been pointed out that gains in a test of intelligence designed to measure between-subject differences do not necessarily imply intelligence gains evaluated within subjects (te Nijenhuis et al., 2007).

Reaching a precise understanding about the nature and meaning of cognitive improvement is a difficult endeavor, but in a field with far-reaching implications for society such as cognitive training, it is worth reflecting upon what training is thought and intended to achieve. Although informed by prior research (e.g., Ellis, 1965; Stankov and Chen, 1988a,b), practicing specific cognitive tasks to elicit transfer is a novel paradigm in its current form, and numerous questions remain regarding the definition and measure of cognitive enhancement (e.g., te Nijenhuis et al., 2007; Moreau, 2014a). Until theoretical models are refined to account for novel evidence, we cannot assume that long-standing knowledge based on more than a century of research in psychometrics applies inevitably to training designs deliberately intended to promote general cognitive improvement.

## Single Transfer Assessments

Beyond matters of analysis and interpretation, the choice of specific tasks used to demonstrate transfer is also critical. Any measurement, no matter how accurate, contains error. More than anywhere else perhaps, this is true in the behavioral sciences—human beings differ from one another on multiple factors that contribute to task performance in any ability. One of the keys to reduce error is to increase the number of measurements. This idea might not be straightforward at first—if measurements are imperfect, why would multiplying them, and therefore the error associated with them, give a better estimate of the ability one wants to probe? The reason multiple measurements are superior to single measurements is because inferring scores from combined sources allows extracting out some, if not most, of the error.

This notion is ubiquitous. Teachers rarely give final grades based on one assessment, but rather average intermediate grades to get better, fairer estimates. Politicians do not rely on single polls to decide on a course of action in a campaign—they combine several of them to increase precision. Whenever precision matters most, we also increase the number of measurements before combining them. In tennis, men play to the best of three sets in most competitions, but to the best of five sets in the most prestigious tournaments, the Grand Slams. The idea is to minimize the noise, or random sources of error, and maximize the signal, or the influence of a true ability, tennis skills in this example.

This is not the unreasoned caprice of picky scientists—by increasing the number of measurements, we do get better estimates of latent constructs. Nobody says it more eloquently than Randy Engle in *Smarter*, a recent bestseller by Hurley (2014): “Much of the things that psychology talks about, you can’t observe. […] They’re constructs. We have to come up with various ways of measuring them, or defining them, but we can’t specifically observe them. Let’s say I’m interested in love. How can I observe love? I can’t. I see a boy and a girl rolling around in the grass outside. Is that love? Is it lust? Is it rape? I can’t tell. But I define love by various specific behaviors. Nobody thinks any one of those in isolation is love, so we have to use a number of them together. Love is not eye contact over dinner. It’s not holding hands. Those are just manifestations of love. And intelligence is the same.”

**Figure 5:**
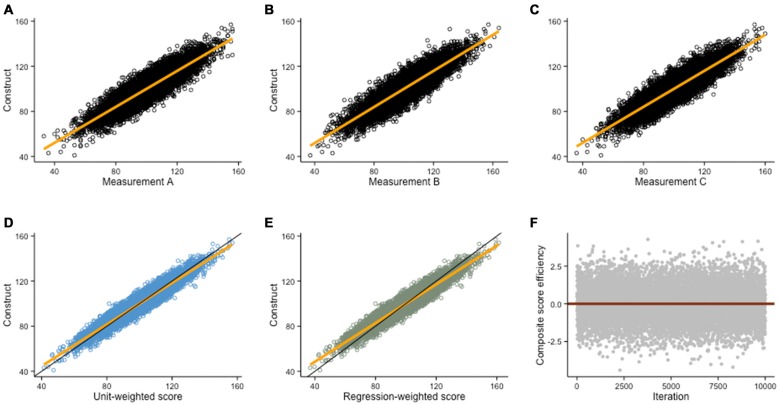
**Composite scores.** Monte Carlo simulation (*N* = 10,000) of three imperfect measurements of a hypothetical construct (construct is normally distributed with *M* = 100 and SD = 15; random error is normally distributed with *M* = 0 and SD = 7.5). **(A–C)** Scatterplots depicting the relationship between the construct and each of its imperfect measurements (*r* = .89, in all cases). **(D)** A better estimate of the construct is given by a unit-weighted composite score (*r* = .96), or by **(E)** a regression-weighted composite score (*r* = .96) based on factor loadings of each measurement in a factor analysis. **(F)** In this example, the difference between unit-weighted scores and regression-weighted is negligible because the correlations of each measurement with the true ability are roughly equal. Thus, observations above the red line (regression-weighted score is the best estimate) and below the red line (unit-weighted score is the best estimate) approximately average to 0.

Because constructs are not directly observable (i.e., latent), we rely on combinations of multiple measurements to provide accurate estimates of cognitive abilities. Measurements can be combined into composite scores, that is, scores that minimize measurement error to better reflect the underlying construct of interest. Because they typically improve both reliability and validity in measurements (Carmines and Zeller, 1979), composite scores are key in cognitive training designs (e.g., Shipstead et al., 2012). Relying on multiple converging assessments also allows adequate scopes of measurement, which ensure that constructs reflect an underlying ability rather than task-specific components (Noack et al., 2014). Such precaution in turn allows stronger and more accurate claims of transfer after an intervention. Again, thinking about this idea with an example is helpful. Suppose we simulate an experiment in which we set to measure intelligence (*g*) in a sample of participants. Defining a construct *g* and three imperfect measures of *g* reflecting the true ability plus normally distributed random noise, we obtain single measures that correlate with *g* such that *r* = .89 (Figures 5A–C). Let us assume three *different* assessments of *g* rather than three consecutive testing sessions of the same assessment, so that we do not need to take testing effects into account.

Different solutions exist to minimize measurement error, besides ensuring experimental conditions were adequate to guarantee valid measurements. One possibility is to use the median score. Although not ideal, this is an improvement over single testing. Another solution is to average all scores and create a unit-weighted composite score (i.e., mean, Figure 5D), which often is a better estimate than the median, unless one or several of the measurements were unusually prone to error. When individual scores are strongly correlated (i.e., collinear), a unit-weighted composite score is often close to the best possible estimate. When individual scores are not or weakly correlated, a regression-weighted composite score is usually a better estimate as it allows minimizing error (Figure 5E). Weights for the latter are factor loadings extracted from a factor analysis that includes each measurement, thus minimizing non-systematic error. The power of composite scores is more evident graphically—Figures 5D,E show how composite scores are better estimates of a construct than either measure alone (including the median, see in comparison with Figures 5A–C). Different methods to generate composite scores can themselves be subsequently compared (see Figure 5F). To confidently claim transfer after an intervention, one therefore needs to demonstrate that gains are not exclusive to single tasks, but rather reflect general improvement on latent constructs.

Directly in line with this idea, more advanced statistical techniques such as latent curve models (LCM) and latent change score models (LCSM), typically implemented in a SEM framework, can allow finer assessment of training outcomes (for example, see Ghisletta and McArdle, 2012, for practical implementation). Because of its explicit focus on change across different time points, LCSM is particularly well suited to the analysis of longitudinal data (e.g., Lövdén et al., 2005) and of training studies (e.g., McArdle, 2009), where the emphasis is on cognitive improvement. Other possibilities exist, such as multilevel (Rovine and Molenaar, 2000), random effects (Laird and Ware, 1982) or mixed models (Dean and Nielsen, 2007), all with a common goal: minimizing noise in repeated-measures data, so as to separate out measurement error from predictors or structural components, thus yielding more precise estimates of change.

## Multiple Comparisons

If including too few dependent variables is problematic, too many can also be prejudicial. At the core of this apparent conundrum lies the multiple comparisons problem, another subtle but pernicious limitation in experimental designs. Following up on one of our previous examples, suppose we are comparing a novel cognitive remediation program targeting learning disorders with traditional feedback learning. Before and after the intervention, participants in the two groups can be compared on measures of reading fluency, reading comprehension, WMC, arithmetic fluency, arithmetic comprehension, processing speed, and a wide array of other cognitive constructs. They can be compared across motivational factors, or in terms of attrition rate. And questionnaires might provide data on extraversion, happiness, quality of life, and so on. For each dependent variable, one could test for differences between the group receiving the traditional intervention and the group enrolled in the new program, with the rationale that differences between groups reflect an inequality of the treatments.

**Figure 6:**
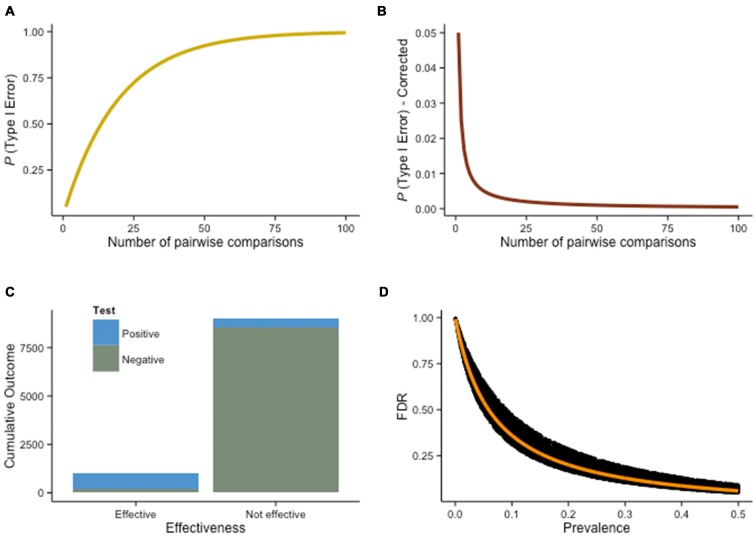
**Multiple comparisons problem. (A)** True probability of a Type I error as a function of the number of pairwise comparisons (holding α = .05 constant for any given comparison). **(B)** A possible solution to the multiple comparisons problem is the Bonferroni correction, a rather conservative procedure—the false discovery rate (FDR) decreases, but so does the true discovery rate, increasing the chance to miss a true difference (β). **(C)** Assuming 10% of training interventions are truly effective (*N* = 10, 000), with power = .80 and α = .05, 450 interventions will yield false positives, while 800 will result in true positives. The FDR in this situation is 36% (more than a third of the positive results are not real), despite high power and standard α criteria. Therefore, this estimate is conservative—assuming 10% of the interventions are effective and 1−β = .80 is rather optimistic. **(D)** As the prevalence of an effect increases (e.g., higher percentage of effective interventions overall), the FDR decreases. This effect is illustrated here with a Monte Carlo simulation of the FDR as a function of prevalence, given 1−β free to vary such that .50 ≤ 1−β ≤ 1 (the orange line shows FDR when 1−β = .80).

With the multiplication of pairwise comparisons, however, experimenters run the risk of finding differences by chance alone, rather than because of the intervention itself.^4^ As we mentioned earlier, mistaking a random fluctuation for a true effect is a false positive, or Type I error. But what exactly is the probability to wrongly conclude that an effect is genuine when it is just random noise? It is easier to solve this problem graphically (Figure 6A). When comparing two groups on 10 transfer tasks, the probability to make a wrong judgment because of random fluctuation is about 40%. With 15 tasks, the probability rise to 54%, and with 20 tasks, it reaches 64% (all assuming a α = .05 threshold to declare a finding significant).

This problem is well known, and procedures have been developed to account for it. One evident answer is to reduce Type I errors by using a more stringent threshold. With α = .01, the percentage of significant differences rising spuriously in our previous scenario drops to 10% (10 tasks), 14% (15 tasks), and 18% (20 tasks). Lowering the significance threshold is exactly what the Bonferroni correction does (Figure 6B). Specifically, it requires dividing the significance level required to claim that a difference is significant by the number of comparisons being performed. Therefore, for the example above with 10 transfer tasks, α = .005, with 15 tasks, α = .003, and with 20 tasks, α = .0025. The problem with this approach is that it is often too conservative—it corrects more strictly than necessary. Considering the lack of power inherent to numerous interventions, true effects will often be missed when the Bonferroni procedure is applied; the procedure lowers false discoveries, but by the same token lowers true discoveries as well. This is especially problematic when comparisons are highly dependent (Vul et al., 2009; Fiedler, 2011). For example, in typical fMRI experiments involving the comparisons of thousands of voxels with one another, Bonferroni corrections would systematically prevent yielding any significant correlation. By controlling α levels across all voxels, the method guarantees an error probability of .05 on each single comparison, a level too stringent for discoveries. Although the multiple comparisons problem has been extensively discussed, we should point out that not everyone agrees on its pernicious effects (Gelman et al., 2012).

Provided there is a problem, a potential solution is replication. Obviously, this is not always feasible, can turn out to be expensive, and is not entirely foolproof. Other techniques have been developed to answer this challenge, with good results. For example, the recent rise of Monte Carlo methods or their non-parametric equivalent such as bootstrap and jackknife offers interesting alternatives. In intervention that include brain imaging data, these techniques can be used to calculate cluster-size thresholds, a procedure that relies on the assumption that contiguous signal changes are more likely to reflect true neural activity (Forman et al., 1995), thus allowing more meaningful control over discovery rates.

In line with this idea, one approach that has gained popularity over the years is based on the false discovery rate (FDR). FDR correction is intended to control false discoveries by adjusting α only in the tests that result in a discovery (true or false), thus allowing a reduction of Type I errors while leaving more power to detect truly significant differences. The resulting *q*-values are corrected for multiple comparisons, but are less stringent than traditional corrections on *p*-values because they only take into account positive effects. To illustrate this idea, suppose 10% of all cognitive interventions are effective. That is, of all the designs tested by researchers with the intent to improve some aspect of cognition, one in 10 is a successful attempt. This is a deliberately low estimate, consistent with the conflicting evidence surrounding cognitive training (e.g., Melby-Lervåg and Hulme, 2013). Note that we rarely know beforehand the ratio of effective interventions, but let us assume here that we do. Imagine now that we wish to know which interventions will turn out to show a positive effect, and which will not, and that α = .05 and power is .80 (both considered standard in psychology). Out of 10,000 interventions, how often will we wrongly conclude that an intervention is effective?

To determine this probability, we first need to determine how many interventions overall will yield a positive result (i.e., the experimental group will be significantly different from the control group at posttest). In our hypothetical scenario, we would detect, with a power of .80, 800 true positives. These are interventions that were effective (*N* = 1000) and would be correctly detected as such (true positives). However, because our power is *only* .80, we will miss 200 interventions (false negatives). In addition, out of the 9000 interventions that we know are ineffective, 5% (α) will yield false positives. In our example, these amount to 450. The true negatives would be the remaining 8550 (Figure 6C).

The FDR is the amount of false positives divided by all the positive results, that is, 36% in this example. More than 1/3 of the positive studies will not reflect a true underlying effect. The positive predictive value (PPV), the probability that a significant effect is genuine, is approximately two thirds in this scenario (64%). This is worth pausing for a moment: more than a third of our positive results, reaching significance with standard frequentist methods, would be misleading. Furthermore, the FDR increases if either power or the percentage of effective training interventions in the population of studies decreases (Figure 6D). Because FDR only corrects for positive *p*-value, the procedure is less conservative than the Bonferroni correction. Many alternatives exist (e.g., Dunnett’s test, Fisher’s LSD, Newman-Keuls test, Scheffé’s method, Tukey’s HSD)—ultimately, the preferred method depends on the problem at hand. Is the emphasis on finding new effects, or on the reliability of any discovered effect? Scientific rationale is rarely dichotomized, but thinking about a research question in these terms can help to decide on adequate statistical procedures. In the context of this discussion, one of the best remedies remains to design an intervention with a clear hypothesis about the variables of interest, rather than multiply outcome measures and increase the rate of false positives. Ideally, experiments should explicitly state whereas they are exploratory or confirmatory (Kimmelman et al., 2014), and should always disclose all tasks used in pretest and posttest sessions (Simmons et al., 2011). These measures are part of a broader ongoing effort intended to reduce false positives in psychological research, via more transparency and systematic disclosure of all manipulations, measurements and analyses in experiments, to control for researcher degrees of freedom (Simmons et al., 2011).

## Publication Bias

Our final stop in this statistical journey is to discuss publication bias, a consequence of research findings being more likely to get published based on the direction of the effects reported or on statistical significance. At the core of the problem lies the overuse of frequentist methods, and particularly H_0_ Significance Testing (NHST), in medicine and the behavioral sciences, with an emphasis on the likelihood of the collected data or more extreme data if the H_0_ is true—in probabilistic notation, *P*(d|H_0_) – rather than the probability of interest, *P*(H_0_|d), that is, the probability that the H_0_ is true given the data collected. In intervention studies, one typically wishes to know the probability that an intervention is effective given the evidence, rather than the less informative likelihood of the evidence if the intervention were ineffective (for an in-depth analysis, see Kirk, 1996). The incongruity between these two approaches has motivated changes in the way findings are reported in leading journals (e.g., Cumming, 2014) punctuated recently by a complete ban of NHST in *Basic and Applied Social Psychology* (Trafimow and Marks, 2015), and is central in the growing popularity of Bayesian inference in the behavioral sciences (e.g., Andrews and Baguley, 2013).

Because of the underlying logic of NHST, only rejecting the H_0_ is truly informative—retaining H_0_ does not provide evidence to prove that it is true^5^. Perhaps the H_0_ is untrue, but it is equally plausible that the strength of evidence is insufficient to reject H_0_ (i.e., lack of power). What this means in practice is that null findings (findings that do not allow us to confidently reject H_0_) are difficult to interpret, because they can be due to the absence of an effect or to weak experimental designs. Publication of null findings is therefore rare, a phenomenon that contribute to bias the landscape of scientific evidence—only positive findings get published, leading to the false belief that interventions are effective, whereas a more comprehensive assessment might lead to more nuanced conclusions (e.g., Dickersin, 1990).

**Figure 7:**
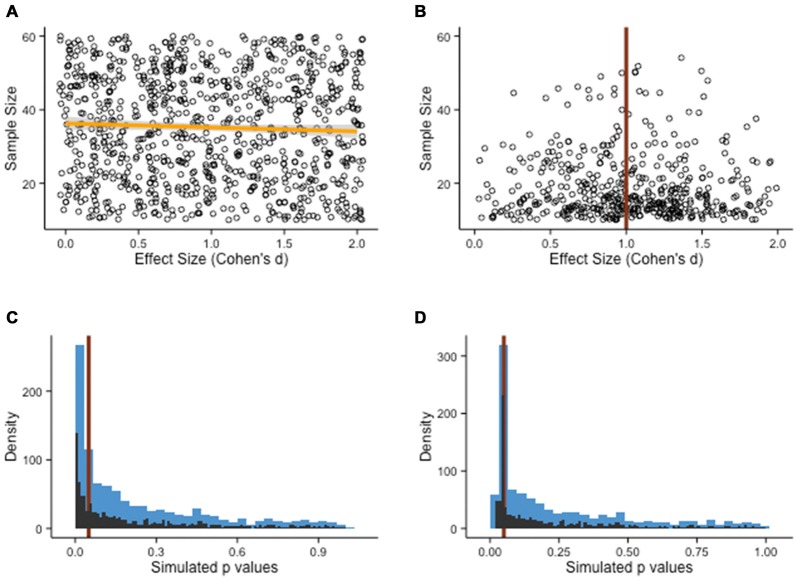
**Publication bias. (A)** Population of study outcomes, with effect sizes and associated sample sizes. There is no correlation between the two variables, as shown by the slope of the fit line (orange line). **(B)** It is often assumed that in the absence of publication bias, the population of studies should roughly resemble the shape of a funnel, with small sample studies showing important variation and large sample studies clustered around the true effect size (in the example, *d* = 1). **(C)** Alternatives have been proposed (see main text), such as the p-curve. Here, a Monte Carlo simulation of two-sample *t*-tests with 1000 draws (*N* = 20 per groups, *d* = 0.5, *M* = 100, SD = 20) shows a shape resembling a power-law distribution, with no particular change around the *p* = .05 threshold (red line). The blue bars represent the rough density histogram, while the black bars are more precise estimates. **(D)** When the pool of published studies is biased, a sudden peak can sometimes be observed just below the *p* = .05 threshold (red line). Arguably, this irregularity in the distribution of *p*-values has no particular justification, if not for bias.

Single studies are never definitive; rather, researchers rely on meta-analyses pooling together all available studies meeting a set of criteria and of interest to a specific question to get a better estimate of the accumulated evidence. If only studies corroborating the evidence for a particular treatment get published, the resulting literature becomes biased. This is particularly problematic in the field of cognitive training, due to the relative novelty of this line of investigation, which increases the volatility of one’s belief, and because of its potential to inform practices and policies (Bossaer et al., 2013; Anguera and Gazzaley, 2015; Porsdam Mann and Sahakian, 2015). Consider for example that different research groups across the world have come to dramatically opposite conclusions about the effectiveness of cognitive training, based on slight differences in meta-analysis inclusion criteria and models (Melby-Lervåg and Hulme, 2013; Karbach and Verhaeghen, 2014; Lampit et al., 2014; Au et al., 2015). The point here is that even a few missing studies in meta-analyses can have important consequences, especially when the accumulated evidence is relatively scarce as is the case in the young field of cognitive training. Again, let us illustrate this idea with an example. Suppose we simulate a pool of study results, each with a given sample size and an observed effect size for the difference between experimental and control gains after a cognitive intervention. The model draws pairs of numbers randomly from a vector of sample size (ranging from *N* = 5 to *N* = 100) and a vector of effect sizes (ranging from *d* = 0 to *d* = 2). We then generate stochastically all kinds of associations, for example large sample sizes with small effect sizes and vice-versa (Figure 7A). In science, however, the landscape of published findings is typically different—studies get published when they pass a test of statistical significance, with a threshold given by the *p*-value. To demonstrate that a difference is significant in this framework, one needs large sample sizes, large effects, or a fairly sizable combination of both. When represented graphically, this produces a funnel plot typically used in meta-analyses (Figure 7B); departures from this symmetrical representation often indicate some bias in the literature.

Two directions seem particularly promising to circumvent publication bias. First, researchers often try to make an estimate of the size of publication bias when summarizing the evidence for a particular intervention. This process can be facilitated by examining a representation of all the published studies, with a measure of precision plotted as a function of the intervention effect. In the absence of publication bias, it is expected that studies with larger samples, and therefore better precision, will fall around the average effect size observed, whereas studies with smaller sample size, lacking precision, will be more dispersed. This results in a funnel shape within which most observations fall. Deviations from this shape can raise concerns regarding the objectivity of the published evidence, although it should be noted that other explanations might be equally valid (Lau et al., 2006). These methods can be improved upon, and recent articles have addressed some of the typical concerns of solely relying on funnel plots to estimate publication bias. Interesting alternatives have emerged, such as *p*-curves (see Figures 7C,D; Simonsohn et al., 2014a,b) or more direct measures of the plausibility of a set of findings (Francis, 2012). These methods are not infallible (for example, see Bishop and Thompson, 2016), but they represent steps in the right direction.

Second, ongoing initiatives are intended to facilitate the publication of all findings, irrespective of the outcome, on online repositories. Digital storage has become cheap, allowing platforms to archive data for limited cost. Such repositories already exist in other fields (e.g., arXiv), but have not been developed fully in medicine and in the behavioral sciences. Additional incentives to pre-register studies are another step in that direction—for example, allowing researchers to get preliminary publication approval based on study design and intended analyses, rather than on the direction of the findings. Publishing all results would eradicate publication bias (van Assen et al., 2014), and therefore initiatives such as pre-registration should be the favored approach in the future (Goldacre, 2015).

## Conclusion

Based on Monte Carlo simulations, we have demonstrated that several statistical flaws undermine typical findings in cognitive training interventions. This critique echoes others, which have pointed out the limitations of current research practices (e.g., Ioannidis, 2005), although arguably the flaws we discussed in this article are often a consequence of limited resources—including methodological and statistical guidelines—rather than the result of errors or practices deliberately intended to mislead. These flaws are pervasive, but we believe that clear visual representations can help raise awareness of their pernicious effects among researchers and interested readers of scientific findings. As we mentioned, statistical flaws are not the only kinds of problems in cognitive training interventions. However, the relative opacity of statistics favors situations where one applies methods and techniques popular in a field of study, irrespective of pernicious effects. We hope that our present contribution provides a valuable resource to make training interventions more accessible.

Importantly, not all interventions suffer from these flaws. A number of training experiments are excellent, with strong designs and adequate data analyses. Arguably, these studies have emerged in response to prior methodological concerns and through facilitated communication across scientific fields, such as between evidence-based medicine and psychology, stressing further the importance of discussing good research practices. One example that illustrates the benefits of this dialog is the use of active control groups, which is becoming the norm rather than the exception in the field of cognitive training. When feasible, other important components are being integrated within research procedures, such as random allocation to conditions, standardized data collection and double-blind designs. Following current trends in the cognitive training literature, interventions should be evaluated according to their methodological and statistical strengths—more value, or weight, should be given to flawless studies or interventions with fewer methodological problems, whereas less importance should be conferred to studies that suffer several of the flaws we mentioned (Moher et al., 1998).

Related to this idea, most simulations in this article stress the limit of frequentist inference in its NHST implementation. This idea is not new (e.g., Bakan, 1966), yet discussions of alternatives are recurring and many fields of study are moving away from solely relying on the rejection of null hypotheses that often make little practical sense (Herzog and Ostwald, 2013; but see also Leek and Peng, 2015). In our view, arguing for or against the effectiveness of cognitive training is ill-conceived in a NHST framework, because the overwhelming evidence gathered throughout the last century is in favor of a null-effect. Thus, even well-controlled experiments that fail to reject the H_0_ cannot be considered as convincing evidence against the effectiveness of cognitive training, despite the prevalence of this line of reasoning in this literature.

As a result, we believe cognitive interventions are particularly suited to alternatives such as Neyman-Pearson Hypothesis Testing (NPHT) and Bayesian inference. These approaches are not free of caveats, yet they provide interesting alternatives to the prevalent framework. Because NPHT allows non-significant results to be interpreted as evidence for the null-hypothesis (Neyman and Pearson, 1933), the underlying rationale of NPHT favors scientific advances, especially in the context of accumulating evidence against the effectiveness of an intervention. Bayesian inference (Bakan, 1953; Savage, 1954; Jeffreys, 1961; Edwards et al., 1963) also seems particularly appropriate in evaluating training findings, given the relatively limited evidence for novel training paradigms and the variety of extraneous factors involved. Initially, limited data is outweighed by prior beliefs, but more substantial evidence eventually overwhelms the prior and lead to changes in belief (i.e., updated posteriors). Generally, understanding human cognition follows this principle, with each observation refining the ongoing model. In his time, Piaget speaking of children as “little scientists” was hinting on this particular point—we construct, update and refine our model of the world at all times, taking into account the available data and confronting them with prior experience. A full discussion of Bayesian inference applications in the behavioral sciences is outside the scope of this article, but many excellent contributions have been published in recent years, either related to the general advantages of adopting Bayesian statistics (Andrews and Baguley, 2013) or introducing Bayesian equivalents to common frequentist procedures (Wagenmakers, 2007; Rouder et al., 2009; Morey and Rouder, 2011; Wetzels and Wagenmakers, 2012; Wetzels et al., 2012). It follows that evidence should not be dichotomized—some interventions work for some individuals, and what needs to be identified is what particular interventions yield the more sizeable or reliable effects, what individuals benefit from these and why, rather than the elusive question of absolute effectiveness (Moreau and Waldie, 2016).

In closing, we remain optimistic about current directions in evidence-based cognitive interventions—experimental standards have been improved (Shipstead et al., 2012; Boot et al., 2013), in direct response to blooming claims reporting post-intervention cognitive enhancement (Karbach and Verhaeghen, 2014; Au et al., 2015) and their criticisms (Shipstead et al., 2012; Melby-Lervåg and Hulme, 2013). Such inherent skepticism is healthy, yet hurdles should not discourage efforts to discover effective treatments. The benefits of effective interventions to society are enormous, and further research is to be supported and encouraged. In line with this idea, the novelty of cognitive training calls for exploratory designs to discover effective interventions. The present article represents a modest attempt to document and clarify experimental pitfalls so as to encourage significant advances, at a time of intense debates sparking around replication in the behavioral sciences (Pashler and Wagenmakers, 2012; Simons, 2014; Baker, 2015; Open Science Collaboration, 2015; Simonsohn, 2015; Gilbert et al., 2016). By presenting common pitfalls and by reflecting on ways to evaluate typical designs in cognitive training, we hope to provide an accessible reference for researchers conducting experiments in this field, but also a useful resource for neophytes interested in understanding the content and ramifications of cognitive intervention studies. If scientists want training interventions to impact decisions outside research and academia, empirical findings need to be presented in a clear and unbiased manner, especially when the question of interest is complex and the evidence equivocal.

## Author Contributions

DM designed and programmed the simulations, ran the analyses, and wrote the paper. IJK and KEW provided valuable suggestions. All authors approved the final version of the manuscript.

## Funding

Part of this work was supported by philanthropic donations from the Campus Link Foundation, the Kelliher Trust and Perpetual Guardian (as trustee of the Lady Alport Barker Trust) to DM and KEW.

## Conflict of Interest Statement

The authors declare that the research was conducted in the absence of any commercial or financial relationships that could be construed as a potential conflict of interest.

## Footnotes

^1^Step (2) was implemented in R (R Core Team, 2014) because of its growing popularity among researchers and data scientists (Tippmann, 2015), and because R is free and open-source, thus allowing anyone, anywhere, to reproduce and build upon our analyses. We used R version 3.1.2 (R Core Team, 2014) and the following packages: ggplot2 (Wickham, 2009), gridExtra (Auguie, 2012), MASS (Venables and Ripley, 2002), MBESS (Kelley and Lai, 2012), plyr (Wickham, 2011), psych (Revelle, 2015), pwr (Champely, 2015), and stats (R Core Team, 2014).

^2^For a given α and effect size, low power results in low Positive Predictive Value (PPV), that is, a low probability that a significant effect observed in a sample reflects a true effect in the population. The PPV is closely related to the False Discovery Rate (FDR) mentioned in the section on multiple comparisons of this article, such that PPV + FDR = 1.

^3^More advanced statistical techniques (e.g., latent change score models) can help to refine claims of transfer in situations where multiple outcome variables are present (e.g. McArdle and Prindle, 2008; McArdle, 2009; Noack et al., 2014).

^4^Multiple comparisons introduce additional problems in training designs, such as practice effects from one task to another within a given construct (i.e., hierarchical learning, Bavelier et al., 2012), or cognitive depletion effects (Green et al., 2014).

^5^David Bakan distinguished between sharp and loose null hypotheses, the former referring to the difference between population means being strictly zero, whereas the latter assumes this difference to be *around* the null. Much of the disagreement with NHST arises from the problem presented by sharp null hypotheses, which, given sufficient sample sizes, are never true (Bakan, 1966).
